# Foliar Zinc Application to Wheat May Lessen the Zinc Deficiency Burden in Rural Quzhou, China

**DOI:** 10.3389/fnut.2021.697817

**Published:** 2021-06-28

**Authors:** Bao-Gang Yu, Yu-Min Liu, Xiu-Xiu Chen, Wen-Qing Cao, Tong-Bin Ding, Chun-Qin Zou

**Affiliations:** Key Laboratory of Plant-Soil Interactions, Ministry of Education, College of Resources and Environmental Sciences, National Academy of Agriculture Green Development, China Agricultural University, Beijing, China

**Keywords:** agronomic biofortification, zinc, wheat, health benefits, DALYs, Quzhou county

## Abstract

Zinc (Zn) malnutrition is a common health problem, especially in developing countries. The human health and economic benefits of the replacement of conventional flour with Zn-biofortified wheat flour in rural household diets were assessed. One hundred forty-five wheat flour samples were collected from rural households in Quzhou County. Then, field experiments were conducted on wheat at two Zn levels (0 and 0.4% ZnSO_4_ · 7H_2_O foliar application) under 16 diverse agricultural practices in Quzhou County. Foliar Zn application significantly increased the Zn concentration and bioavailability in wheat grain and flour. If rural households consumed Zn-biofortified flour instead of self-cultivated flour or flour purchased from supermarkets, 257–769 or 280–838, 0.46–1.36 million or 0.50–1.49 million disability-adjusted life years (DALYs) lost, respectively, could be saved in Quzhou County and China. Amounts of 2.3–12.0 million and 5.5–22.6 billion RMB could be obtained via Zn-biofortified flour in Quzhou County and China, respectively. The current study indicates that Zn-biofortified flour via foliar Zn application is a win-win strategy to maintain the yield and combat human Zn deficiency in rural households in China. More health and economic benefits could be obtained in rural household dependent on wheat flour purchased from supermarkets than in those dependent on self-cultivated wheat flour.

## Introduction

As an essential micronutrient, zinc (Zn) plays a vital role in crop production and human nutrition. At present, Zn deficiency, also called hidden hunger, is a common public issue worldwide, contributing to many health problems (1). Zn deficiency is one of the five leading risk factors resulting in disease and death, and one-third of the global population suffers from Zn malnutrition (2). In China, more than 86 million people suffered from an insufficient Zn intake, and the development of 10 million children (<5 years) was stunted (3).

The widespread occurrence of Zn malnutrition in humans mainly arises from a low dietary intake of Zn (4). Currently, cereal crops are a major dietary source of calories, protein, and Zn worldwide, especially in developing countries (5). However, approximately half of the soils in cereal cultivation is Zn deficient, resulting in an inadequate Zn content in cereal foods to satisfy the human demand (1).

As one of the three major cereal crops, China ranks at the top in terms of the cultivation area and annual production of wheat globally, and wheat is widely used in staple foods and livestock feed, especially in rural areas (6). However, the average Zn concentration in wheat grain is only 23.3 mg kg^−1^ in China, indicating a wide gap to the target value of 40 mg kg^−1^ (7). In addition, the Zn concentration in wheat flour is positively correlated with the Zn concentration in wheat grain. However, during grain milling, most Zn is typically lost and bound to phytic acid (PA), which further leads to a marked reduction in the Zn intake (8). Therefore, there is an urgent need to improve the Zn concentration and bioavailability in wheat grain and flour to minimize Zn malnutrition.

In recent years, nutritionists have proposed many strategies to overcome Zn deficiency, such as dietary diversification, food fortification, and supplementation. However, these strategies are difficult to implement in developing countries due to the high cost and other social reasons (9). Biofortification-a new strategy to improve the micronutrient contents in edible parts-is potentially more applicable than are other strategies (1). Previous studies have suggested that agronomic biofortification (i.e., fertilization) is a highly cost-effective strategy to improve human health in the short term over genetic biofortification (10). Among agronomic biofortification techniques, it has been well-established, based on a variety of studies, that foliar Zn application is a much more effective method than soil Zn application in Zn concentration enhancement, and the increase in the Zn concentration in wheat grain and flour via foliar Zn application is nearly 2-fold (11, 12). However, many studies have primarily focused on a given field condition, and there may be a higher practical significance to analyze the effects of foliar Zn application on the Zn concentration and bioavailability in wheat grain and flour under diverse agricultural practices (soil properties, wheat cultivar, fertilization, and management).

Quzhou County is located in Handan city, Hebei Province, China, and 97% of the total population in Quzhou County is engaged in agriculture (13). The cultivated land area of winter wheat is 216.8 km^2^ in Quzhou County (41% of the total cultivated land area), consisting of 10 townships and 342 administrative villages. However, a previous survey has revealed that 39% of the children in Handan city suffers from Zn malnutrition (14). As an important county of wheat production in Handan city, the Zn deficiency value could increase in Quzhou County, especially in rural households mainly consuming self-cultivated wheat, due to the low soil DTPA-Zn concentration (15) and relatively high phosphorus (P) fertilization level (16). Therefore, it is feasible and meaningful to study the health impact of the agronomic Zn biofortification of wheat in Quzhou County.

The objectives of this study were (1) to analyze the current Zn intake based on samples of the wheat flour consumed daily in rural households in Quzhou County, (2) to study the effects of foliar Zn application on the Zn concentration and bioavailability in wheat grain and flour under diverse agricultural practices in Quzhou County, and (3) to comprehensively assess the health and economic impacts of the replacement of conventional wheat flour with Zn-biofortified flour in rural household diets in Quzhou County and China via scenario simulation.

## Materials and Methods

### Field Locations and Experimental Design

**Figure 1:**
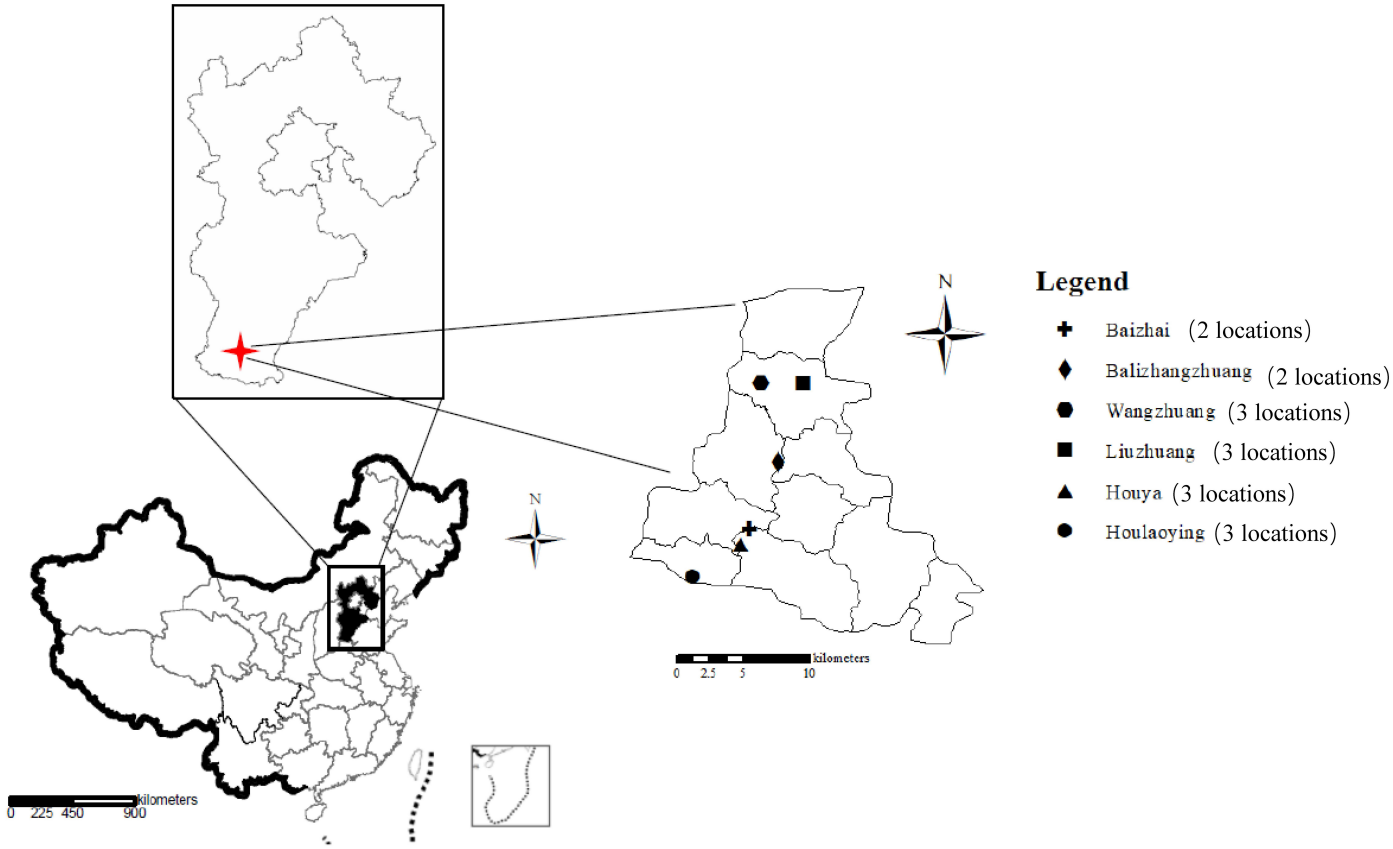
The locations where the field experiments were conducted in Quzhou County, Hebei Province of China. Sixteen experimental locations are located in the 6 villages in Quzhou County.

Field experiments were conducted at 16 locations in Quzhou County (114°50′30″E−115°13′30″E, 36°34′45″N−36°57′57″N) in the North China Plain (Figure 1). Quzhou County contains a typical calcareous alluvial soil and has a subtropical humid monsoon climate, with an average annual temperature and precipitation of 13.4°C and 534.9 mm, respectively (13). Information on the NPK fertilizers, soil properties, and wheat cultivars used at each location is listed in Supplementary Table 1. The winter wheat-summer maize rotation system was applied at all 16 locations.

Each location included two treatments: the control treatment (conventional farmer practices with no Zn application) and foliar Zn application (0.4% ZnSO_4_ · 7H_2_O, w/v). Foliar Zn was applied twice as follows: the first spraying was conducted at the early milk stage, and the second spraying occurred a week later. A Tween (0.01%, v/v) solution was applied at 800 L ha^−1^ in the foliar Zn application treatment. Spraying was conducted on cloudy days or after sunset under windless conditions across all 16 locations. The area of each plot was 100 m^2^ at all 16 locations. In addition to foliar Zn application, routine cropping practices were implemented in the present study, including various seeding rates, fertilizer application, herbicide sprays, and irrigation.

### Sample Collection and Analysis

At maturity in June 2019, 3 of 3-m^2^ area in each plot of grains were collected to determine the yield and nutrient concentrations. The harvested wheat grain samples were washed three times with tap and distilled water. A subsample was retrieved from each treatment, oven dried at 60–65°C to a constant weight and ground into powder with a stainless-steel grinder. Another wheat grain subsample was milled into flour with a Buhler experimental mill (MLU 220, Uzvil, Switzerland), and the rate of flour extraction was ~75%, which is similar to the general flour extraction rate in China market (17).

A total of 145 wheat flour samples was also randomly collected from 21 villages (including the 16 test locations) to estimate the current Zn intake via wheat flour consumption in the rural households in Quzhou County. We divided the sources of wheat flour into two groups: (1) flour milled from wheat grain that was self-cultivated by the rural households (*n* = 124). (2) Flour purchased from the supermarket by the rural households (*n* = 21). These two wheat flour types were separately consumed by the rural households in Quzhou County as part of their daily diets.

The wheat grain and flour samples were digested with 6 mL of HNO_3_ and 2 mL of H_2_O_2_ in a microwave-accelerated reaction system (CEM, Matthews, NC, USA). The micronutrient concentrations in the digested solutions were determined via inductively coupled plasma optical emission spectroscopy (ICP-OES, OPTIMA 3300 DV, Perkin-Elmer, USA). Standard wheat grain material (IPE182) was acquired from the Wageningen Evaluation Programs for Analytical Laboratories (WEPAL, Wageningen University, the Netherlands) and used to ensure consistency and quality. The PA concentrations in the wheat grain and flour samples were analyzed according to a previous study (18). Soil samples (0–20 cm) were also collected at each location to analyze the pH and available Zn and P concentrations after air drying and passing through a 1-mm plastic sieve. The soil pH (water/soil, 2.5:1) was determined with a pH meter (PB-10, Sartorius, GER) (19). The soil available Zn concentration (DTPA-Zn) was analyzed via ICP-OES (OPTIMA 3300 DV, Perkin-Elmer, USA) after extraction with 5 mmol L^−1^ diethylene triamine pentaacetic acid (DTPA) (20). The soil available P (Olsen-P) concentration was measured according Olsen (21).

### Estimated Zn Bioavailability

A trivariate mathematical model of Zn absorption was adopted to predict the Zn bioavailability (22):

$$\begin{array}{l} TAZ=\;0.5\times 65\times 100\times \{A_{MAX}+TDZ+K_{R}\times \left(1+\frac{TDP}{K_{P}}\right)\\ -\sqrt{{\left(A_{MAX}+TDZ+K_{R}\times \left(1+\frac{TDP}{K_{P}}\right)\;\right)}^{2}-4\times A_{MAX}\times TDZ\;}\} \end{array}$$

where TAZ is the total daily absorbed Zn (mg Zn d^−1^), TDZ and TDP are the total daily dietary Zn (mmol Zn day^−1^) and PA (mmol PA day^−1^), respectively, AMAX is the maximum Zn absorption (0.091), KR is the equilibrium dissociation constant of the Zn-receptor binding reaction (0.680), and KP is the equilibrium dissociation constant of the Zn-PA binding reaction (0.033) (23), while the TAZ model is based on daily wheat grain and flour consumption (300 g day^−1^) as the sole source of Zn and phytate for adults (24), which is referred to as the estimated Zn bioavailability.

### Potential Health and Economic Benefits of Zn-Biofortified Wheat Flour

The framework of the disability-adjusted life years (DALYs) is an *ex ante* assessment tool to estimate the burden of micronutrient malnutrition and the health impact of micronutrient-biofortified wheat flour (25). The current health burden (the DALYs lost) was calculated based on a previous study (26). Infants numbered ~0.58 thousand and 13.8 million, and children (1–5 years old) numbered 3.8 thousand and 76.5 million in Quzhou County and China, respectively (27). The total DALYs lost (infants and children) due to human Zn deficiency in Quzhou County and China were 0.2 thousand and 3.7 million years, respectively. The potential health benefits (the DALYs saved) of Zn-biofortified wheat flour were calculated with a modified method based on the increased Zn bioavailability rather than the increased Zn concentration (28). The status quo of the daily Zn intake was 4.90 and 6.00 mg day^−1^ for infants and children, respectively (29). Based on the daily consumption level of wheat flour of 300 g d^−1^ for Chinese adults, infants consume 75 g each day, and children consume 150 g each day (28). The daily Zn intake through Zn-biofortified wheat flour was calculated as the sum of the current daily Zn intake and the increased TAZ level. We assumed that Zn-biofortified wheat flour replaced the self-cultivated flour or the flour purchased by rural households, and no other dietary aspects were changed. Two coverage rates (20% under a pessimistic scenario and 60% under an optimistic scenario) were defined in this study. To simulate the potential impact of the agronomic Zn biofortification of wheat flour in Quzhou County and China, the health benefits (the DALYs saved) of biofortified wheat flour were calculated via the method of Steur et al. (26).

The following equation was adopted to calculate the economic benefit of Zn-biofortified wheat flour:

Economic benefit = total DALYs saved × PCNI – Zn fertilizer cost.

where PCNI is the per capita net income of China based on a previous study (30). Pesticide foliar spraying is a common practice in wheat cropping systems in China, and the effect of foliar Zn application combined with pesticide spraying on the grain Zn concentration is similar to the effect of foliar Zn application alone, which could greatly reduce the labor requirements (31). Hence, in the current study, we only considered the Zn fertilizer cost (90 RMB ha^−1^) according to Wang et al. (31).

### Statistical Analysis

Excel 2010 (Microsoft, USA) and SPSS software (version 26.0) were used for the calculations and statistical analysis. The effects of foliar Zn application on the Zn and PA concentrations and the estimated Zn bioavailability in wheat grain and flour were assessed via one-way analysis of variance (ANOVA) followed by independent *t*-tests (*P* < 0.05). Similarly, the average of the above three parameters (the Zn and PA concentrations and the estimated Zn bioavailability) for the two sources of wheat flour collected from rural households were also compared via independent *t*-tests (*P* < 0.05).

## Results

### Grain Yield

**Figure 2:**
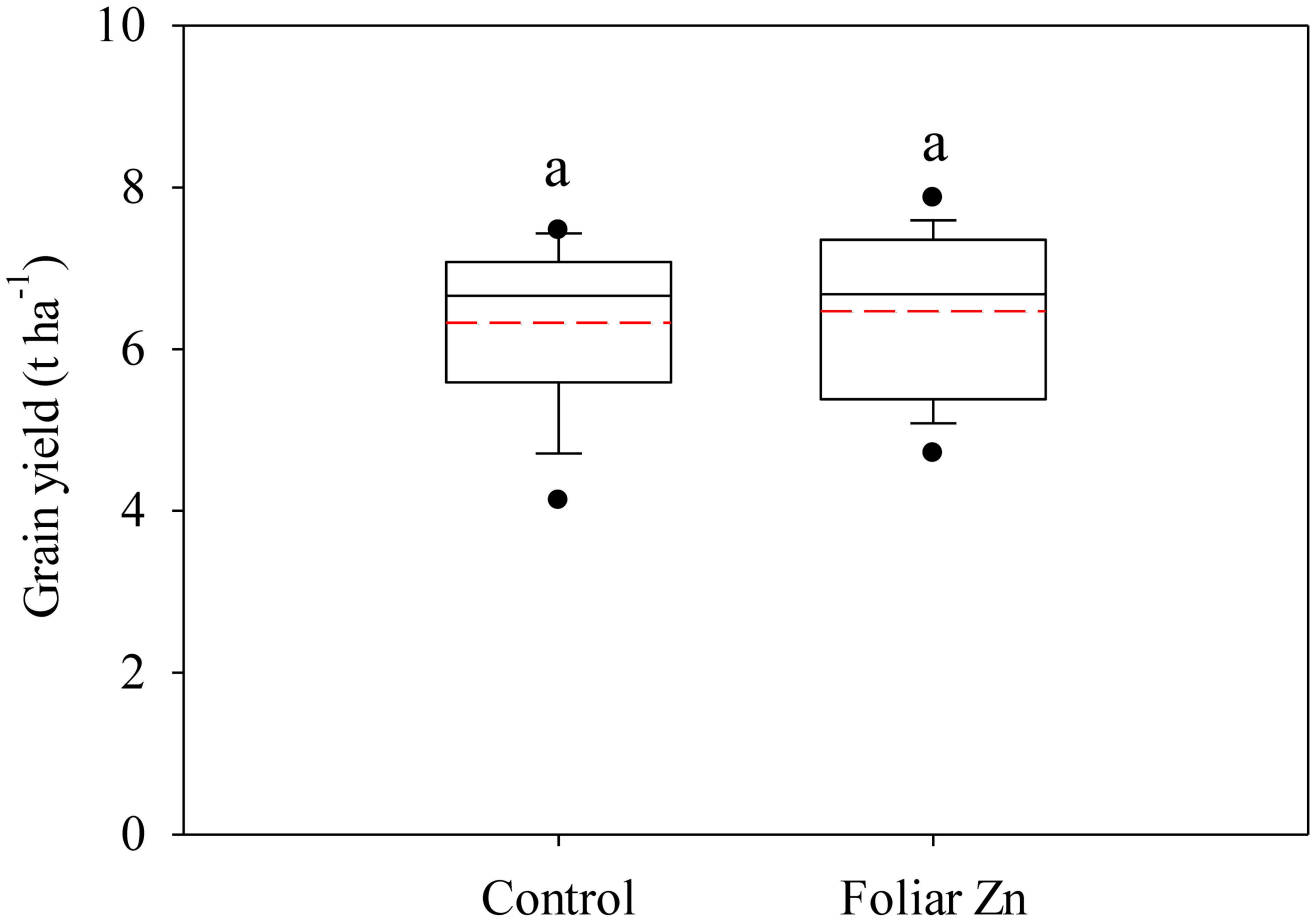
Grain yield (14% water) as affected by foliar Zn application in Quzhou County. The solid and red dashed lines indicate the median and mean values, respectively. The same lowercase letter indicates no significant difference between the foliar Zn application levels via independent *t-*tests at the *P* < 0.05 level.

Foliar Zn application imposed no significant effects on the wheat grain yield at any location (Figure 2). The average grain yields were 6.3 and 6.5 t ha^−1^ under the control and foliar Zn treatments, respectively.

### Zinc and PA Concentrations and Estimated Zn Bioavailability in the Wheat Flour Collected From Rural Households

There was a large variation in the Zn and PA concentrations and estimated Zn bioavailability in the wheat flour samples irrespective of the source. The Zn concentration in the self-cultivated wheat flour was significantly higher than that in the wheat flour purchased from supermarkets. However, no significant differences occurred in the PA concentration and estimated Zn bioavailability between the two sources of wheat flour (Table 1).

**Table 1:** Zinc (Zn) and phytic acid (PA) concentrations and estimated Zn bioavailability in the wheat flour collected from the rural households in Quzhou County.

**Parameters**	**Source of flour**	**Sample number**	**Minimum**	**Maximum**	**Median**	**Mean^a^**	**Coefficient of variation (%)**
Zn concentration (mg kg^−1^)	Self-cultivated	124	4.03	10.94	8.34	8.28a	15.4
	Supermarkets	21	4.56	8.23	7.02	7.05b	13.3
PA concentration (g kg^−1^)	Self-cultivated	124	1.30	3.93	2.83	2.79a	24.3
	Supermarkets	21	1.37	3.74	2.57	2.51a	26.8
Estimated Zn bioavailability (mg Zn d^−1^)	Self-cultivated	124	0.59	1.57	0.85	0.86a	21.2
	Supermarkets	21	0.52	1.30	0.75	0.79a	27.9

a *Means with the same letters indicate no significant difference between two sources of flour at the P < 0.05 level via independent t-tests*.

### Zinc and PA Concentrations and Estimated Zn Bioavailability in the Wheat Grain and Flour Obtained From the Field Experiment

**Figure 3:**
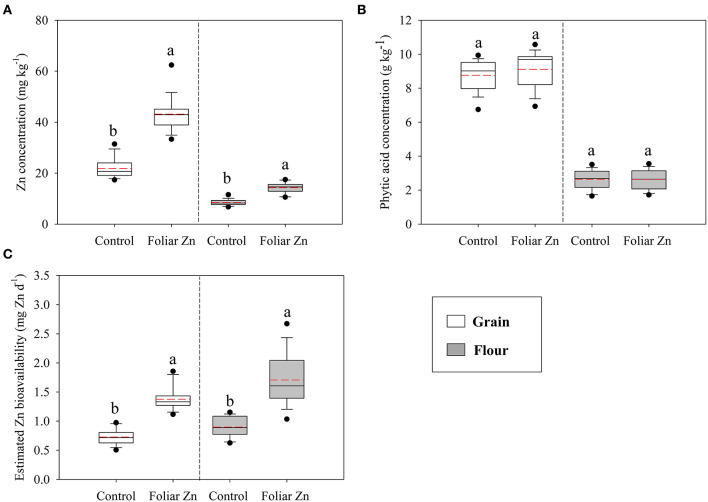
Zinc (Zn) **(A)** and phytic acid **(B)** concentrations and estimated Zn bioavailability **(C)** in wheat grain and flour as affected by foliar Zn application in Quzhou County. The solid and red dashed lines indicate the median and mean values, respectively. The same lowercase letter indicates no significant difference between the foliar Zn application levels via independent *t*-tests at the *P* < 0.05 level. The rate of flour extraction was around 75%.

Without Zn application, the average Zn concentration in wheat grain and flour was 21.8 and 8.5 mg kg^−1^, respectively (Figure 3A). Foliar Zn application significantly increased the Zn concentration in wheat grain and flour. On average, the increases in the Zn concentration in wheat grain and flour caused by foliar Zn application were 97.7 and 68.2%, respectively (Figure 3A). Among the 16 experimental locations, the target grain Zn concentration (40 mg kg^−1^) was obtained at 12 field locations due to foliar Zn application. Foliar Zn application imposed no significant effects on the PA concentration in wheat grain and flour, and the PA concentration in wheat grain was much higher than that in flour across the 16 locations (Figure 3B). Foliar Zn application also significantly increased the Zn bioavailability in wheat grain and flour (Figure 3C). On average, the estimated Zn bioavailability in wheat grain and flour increased from 0.73 to 1.38 mg Zn d^−1^ and from 0.90 to 1.71 mg Zn d^−1^, respectively, via foliar Zn application, resulting in 1.89-fold and 1.90-fold increases, respectively (Figure 3C).

### Health and Economic Impacts of Zn-Biofortified Wheat Flour in Quzhou County and China

Compared to the self**-**cultivated wheat flour or the wheat flour purchased by rural households, Zn-biofortified flour increased the daily Zn intake, the percentage of the recommended intake, the DALYs saved for both infants and children, and the potential economic income (Table 2). Under the pessimistic scenario (20% coverage rate of Zn-biofortified wheat flour), compared to the self-cultivated flour or the flour purchased by rural households, Zn-biofortified wheat flour reduced 12.93 or 14.09% and 12.33 or 13.43% of the current health burden in Quzhou County and China, respectively (Table 2). Under the optimistic scenario (60% coverage rate of Zn-biofortified wheat flour), in contrast to the self-cultivated flour or the flour purchased by rural households, Zn-biofortified wheat flour also saved the current health burden in Quzhou County and China, ranging from 38.67 to 42.15% and 38.71 to 40.18%, respectively (Table 2).

**Table 2:** Health and economic impacts of Zn-biofortified wheat flour on the rural households in Quzhou County and China.

**Parameters**		**Flour from Self-cultivated**		**Flour from supermarket**	
		**Pessimistic scenario**	**Optimistic scenario**	**Pessimistic scenario**	**Optimistic scenario**
**Daily Zn intake (mg day**^**−1**^**, status quo)**					
Infants		4.90	4.90	4.90	4.90
Children		6.00	6.00	6.00	6.00
**Daily Zn intake with foliar Zn application (mg day**^**−1**^**)**					
Infants		5.66	5.66	5.76	5.76
Children		6.76	6.76	6.86	6.86
**% of recommended nutrition intake with foliar Zn application (RNI)**					
Infants		82.03	82.03	83.48	83.48
Children		84.50	84.50	85.75	85.75
**Health impact (“disability-adjusted life years” saved)**					
Quzhou	Infants	40	120	44	131
	Children	217	649	237	707
	Total	257	769	280	838
	% reduction in the current health burden	12.93	38.67	14.09	42.15
China	Infants	117,924	353,772	128,389	385,168
	Children	338,208	1,010,403	368,445	1,101,391
	Total	456,132	1,364,175	496,835	1,486,560
	% reduction in the current health burden	12.33	36.87	13.43	40.18
**Economic impact (RMB)**					
Quzhou		2.3E+06	1.1E+07	2.7E+06	1.2E+07
China		5.5E+09	2.1E+10	6.1E+09	2.3E+10

## Discussion

### Zinc Concentration and Bioavailability in Wheat Flour Consumed by the Rural Households in Quzhou County

In the current study, the Zn concentration in the wheat flour cultivated by farmers exhibited a large variation, which was consistent with Ashin et al. (32). The variations are attributed to the different agricultural management practices, yields, soil fertilities, wheat cultivars, etc. The average Zn concentration in the wheat flour self-cultivated by the rural households in Quzhou County was 8.3 mg kg^−1^ (Table 1). This value was obviously lower than the results of Wang et al. (33). A possible reason for the relatively low Zn concentration in the wheat flour self-cultivated by the rural households in Quzhou County may be the low soil DTPA-Zn concentration (average: 0.82 mg kg^−1^) (15) and high P application level (average: 62.4 kg P ha^−1^) (16), which limits the uptake, translocation, and remobilization of Zn to wheat grain (28, 34). Wei and Cen (35) reported that the average Zn concentration in wheat flour purchased from supermarkets was 5.4 mg kg^−1^ (n = 188), which was lower than our result of 7.1 mg kg^−1^. Different manufacturers and production and processing methods may explain this difference. In addition, the current study demonstrated that the Zn concentration in the flour purchased from supermarkets was lower than that in the flour self**-**cultivated by the rural households. The reason for this may be that the extraction rate of the flour purchased from supermarkets is lower than that of the flour self-cultivated by rural households, and more Zn is lost during milling (36).

Considering Zn homeostasis in human intestines, the Zn bioavailability in wheat flour is more important than the Zn concentration in wheat flour. The observed difference in wheat flour Zn bioavailability between the self-cultivated flour and the flour purchased by rural households suggests a relatively high vulnerability to Zn malnutrition of rural households dependent on wheat flour purchased from supermarkets (Table 1). Rosado et al. (24) reported that 3 mg Zn from the consumption of 300 g wheat flour is the target level for human health. In the present survey, 300 g of the self-cultivated wheat flour and the wheat flour purchased by rural households provided only ~28.7 and 26.3%, respectively, of the daily Zn requirement in Quzhou County.

### Zn-Biofortified Wheat in the Field Experiment

In the present study, foliar Zn application imposed no significant effect on the wheat grain yield, which is consistent with previous studies (37, 38). In good agreement with previous results (11, 37), foliar Zn fertilizer application successfully increased the Zn concentration in wheat grain and flour at all farmer field locations. The increase in the grain Zn concentration due to foliar Zn application is above 3-fold in Iran (39) and Turkey (40). The relatively small increase in the grain Zn concentration (1.98-fold) due to foliar Zn application in the current study may be attributed to the lower soil DTPA-Zn concentration in the above studies than that in the current study at the 16 locations. In addition, the climate conditions, wheat cultivars, and spraying period may also directly affect the extent of the Zn concentration increase in wheat grain and flour (37). Irrespective of foliar Zn application, the highest Zn concentrations in wheat grain and flour were observed in Balizhangzhuang-II, which could be explained by the higher soil DTPA-Zn concentration and higher nitrogen (N) application level (synergistic effect between N and Zn) in Balizhangzhuang-II than those at the other locations (Supplementary Table 1). In addition, in agreement with the results reported by Hussain et al. (41) and Zou et al. (38), the average grain Zn concentration at the 16 locations increased to 43.1 mg kg^−1^ due to foliar Zn application, which matches the biofortification target of Zn in wheat grain.

As a store of P and energy, PA plays an important role in plant growth and development and functions as an antioxidant and anticarcinogen in the human body (42). Unfortunately, to a certain extent, PA is thought to be an antinutrient that reduces the bioavailability of micronutrients, especially iron and Zn (43). In the current study, the PA concentrations in wheat grain and flour remained unchanged in response to foliar Zn application at all locations, which is consistent with Wang et al. (44). The main reason may be that foliar Zn application did not significantly affect the wheat grain yield at any of the 16 locations in the current study (Figure 2).

The current study suggested that the estimated Zn bioavailability in wheat grain and flour was significantly enhanced by foliar Zn application, which is consistent with the findings of Li et al. (45). Based on the target level of 3 mg Zn obtained from the consumption of 300 g wheat flour (24), the estimated Zn bioavailability in wheat grain and flour in the current study is below this level, and 300 g Zn-biofortified wheat flour could provide ~57% of the daily Zn requirements. These results indicate that agronomic biofortification (foliar Zn application) in combination with genetic biofortification (breeding of Zn-efficient genotypes) could be a better choice to minimize Zn deficiency in rural households in future research (7). Our results also showed that the estimated Zn bioavailability in wheat flour was higher than that in wheat grain, which is consistent with published results (46, 47). A possible explanation for these results may be the very low PA concentration in wheat endosperm (48). In addition, as cited in the previous paragraphs, a high application level of P fertilizers is a typical phenomenon under the current wheat cropping management practices in Quzhou County, and combined with the results of the field experiment conducted in Quzhou County, there could be another option to increase the Zn bioavailability in wheat grain and flour via P application optimization (49).

### Health Impact of Zn-Biofortified Wheat Flour in Quzhou County and China

Considering the role of wheat flour as a staple food in Quzhou County and the estimated Zn bioavailability in Zn-biofortified flour being significantly higher than that in the self-cultivated flour or the flour purchased by rural households, substitution of conventional wheat flour with Zn-biofortified flour in rural household diets could highly alleviate Zn malnutrition.

In the current study, the estimated health impact of Zn-biofortified wheat flour was calculated within the DALY framework. Our results indicated that the human health impact (the DALYs saved) in Quzhou County was greater if Zn-biofortified flour replaced the flour purchased from supermarkets than that if the flour produced on rural household farmlands was replaced. This occurs because the Zn concentration and bioavailability in the flour produced on rural household farmlands were higher than those in the flour purchased from supermarkets. Our results also revealed that the reduction in the burden of Zn deficiency in China (12.33–13.43% under the pessimistic scenario and 36.87–40.18% under the optimistic scenario) due to foliar Zn application was larger than that due to biofortification in India (2–12%) and Pakistan (5–33%) (25, 50). In addition, based on the large area of wheat cultivation in Quzhou County (21.7 thousand ha^−1^) (51) and China (23.7 million ha^−1^) (52), a relatively high economic income could be obtained via Zn-biofortified flour in Quzhou County and China, which is consistent with the results of Wang et al. (31). In summary, our results indicate that compared to the flour consumed in rural households on a daily basis, Zn-biofortified wheat flour via foliar Zn application is a feasible strategy to combat human Zn deficiency and potentially increase the economic income in rural households in China.

## Conclusion

Our farm field experiment demonstrated that foliar Zn application effectively increased the Zn concentration and bioavailability in wheat grain and flour irrespective of the agricultural management practices in Quzhou County. Zn-biofortified wheat flour provided ~57% of the daily Zn requirement. Based on the defined scenarios, more health and economic benefits could be obtained by the replacement of self-cultivated flour or flour purchased from supermarkets with Zn-biofortified wheat flour in Quzhou County and China. Therefore, foliar Zn application is a win-win agronomic strategy to maintain the yield and combat human Zn deficiency in rural households in China.

## Data Availability Statement

The raw data supporting the conclusions of this article will be made available by the authors, without undue reservation.

## Author Contributions

B-GY: resources, data curation, and writing—original draft. Y-ML, X-XC, W-QC, and T-BD: investigation. C-QZ: conceptualization, writing—review, and editing. All authors contributed to the article and approved the submitted version.

## Conflict of Interest

The authors declare that the research was conducted in the absence of any commercial or financial relationships that could be construed as a potential conflict of interest.
