# Supplementary material for: Foliar Zinc Application to Wheat May Lessen the Zinc Deficiency Burden in Rural Quzhou, China
Source: Front Nutr. 2021 Jun 28;8:697817. doi: 10.3389/fnut.2021.697817 (PMC8273279; doi:10.3389/fnut.2021.697817)
Supplement: Supplementary file 1 [file Table_1.docx]

**Supplementary Table S1** Information of the NPK fertilizers, soil properties, and wheat cultivars used at each location in Quzhou County

| Location | Soil properties | | | Cultivar | Total fertilizer applied  (kg ha^-1^) | | |
| --- | --- | --- | --- | --- | --- | --- | --- |
|  | pH | DTPA-Zn  (mg kg^-1^) | Olsen-P  (mg kg^-1^) |  | N | P | K_2_O |
| Baizhai-I | 7.8 | 0.95 | 17.2 | Luyuan502 | 225 | 60 | 80 |
| Baizhai-II | 8.0 | 0.72 | 37.9 | Luyuan502 | 240 | 75 | 75 |
| Balizhangzhuang-I | 7.8 | 0.74 | 33.7 | Sankang1 | 300 | 75 | 60 |
| Balizhangzhuang-II | 7.9 | 1.15 | 14.1 | Nongda399 | 220 | 65 | 60 |
| Houlaoying-I | 7.9 | 0.72 | 26.4 | Sankang1 | 230 | 85 | 80 |
| Houlaoying-II | 8.0 | 0.78 | 32.7 | Liangxing99 | 275 | 75 | 85 |
| Houlaoying-III | 8.0 | 0.62 | 47.4 | Sankang1 | 250 | 80 | 75 |
| Houya-I | 7.8 | 0.73 | 29.0 | Lunxuan169 | 300 | 75 | 75 |
| Houya-II | 7.9 | 0.71 | 44.2 | Han6172 | 242 | 80 | 80 |
| Houya-III | 7.9 | 0.64 | 44.0 | Luyuan502 | 275 | 80 | 80 |
| Liuzhuang-I | 7.9 | 0.83 | 20.6 | Liangxing99 | 280 | 70 | 87 |
| Liuzhuang-II | 8.1 | 0.55 | 40.2 | Lunxuan169 | 225 | 80 | 75 |
| Liuzhuang-III | 7.9 | 0.86 | 17.8 | Liangxing99 | 250 | 65 | 75 |
| Wangzhuang-I | 8.1 | 0.65 | 44.6 | Liangxing99 | 265 | 80 | 75 |
| Wangzhuang-II | 7.9 | 0.68 | 31.7 | Liangxing99 | 270 | 75 | 80 |
| Wangzhuang-III | 7.8 | 0.76 | 33.1 | Luyuan502 | 265 | 75 | 75 |


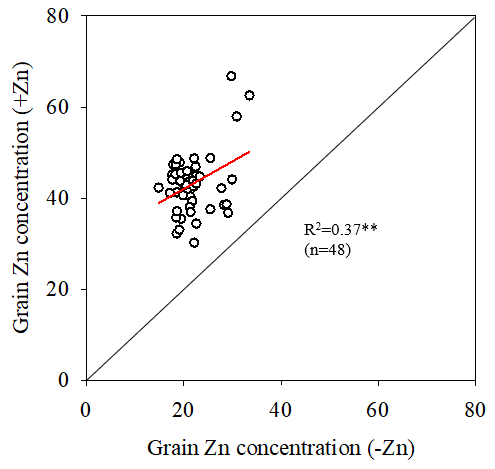


**Supplementary Fig. S1** Linear regression between grain Zn concentration without and with foliar Zn application cross all locations.
